# The Duration of *Chlamydia muridarum* Genital Tract Infection and Associated Chronic Pathological Changes Are Reduced in IL-17 Knockout Mice but Protection Is Not Increased Further by Immunization

**DOI:** 10.1371/journal.pone.0076664

**Published:** 2013-09-20

**Authors:** Dean W. Andrew, Melanie Cochrane, Justin H. Schripsema, Kyle H. Ramsey, Samantha J. Dando, Connor P. O’Meara, Peter Timms, Kenneth W. Beagley

**Affiliations:** 1 Institute of Health and Biomedical Innovation, Queensland University of Technology, Kelvin Grove, Queensland, Australia; 2 Microbiology and Immunology Department, Chicago College of Osteopathic Medicine, Midwestern University, Downers Grove, Illinois, United States of America; University of Texas at San Antonio, United States of America

## Abstract

IL-17 is believed to be important for protection against extracellular pathogens, where clearance is dependent on neutrophil recruitment and local activation of epithelial cell defences. However, the role of IL-17 in protection against intracellular pathogens such as *Chlamydia* is less clear. We have compared (i) the course of natural genital tract *C. muridarum* infection, (ii) the development of oviduct pathology and (iii) the development of vaccine-induced immunity against infection in wild type (WT) BALB/c and IL-17 knockout mice (IL-17-/-) to determine if IL-17-mediated immunity is implicated in the development of infection-induced pathology and/or protection. Both the magnitude and duration of genital infection was significantly reduced in IL-17-/- mice compared to BALB/c. Similarly, hydrosalpinx was also greatly reduced in IL-17-/- mice and this correlated with reduced neutrophil and macrophage infiltration of oviduct tissues. Matrix metalloproteinase (MMP) 9 and MMP2 were increased in WT oviducts compared to IL-17-/- animals at day 7 post-infection. In contrast, oviducts from IL-17-/- mice contained higher MMP9 and MMP2 at day 21. Infection also elicited higher levels of *Chlamydia* -neutralizing antibody in serum of IL-17-/- mice than WT mice. Following intranasal immunization with *C. muridarum* Major Outer Membrane Protein (MOMP) and cholera toxin plus CpG adjuvants, significantly higher levels of chlamydial MOMP-specific IgG and IgA were found in serum and vaginal washes of IL-17-/- mice. T cell proliferation and IFNγ production by splenocytes was greater in WT animals following *in vitro* re-stimulation, however vaccination was only effective at reducing infection in WT, not IL-17-/- mice. Intranasal or transcutaneous immunization protected WT but not IL-17-/- mice against hydrosalpinx development. Our data show that in the absence of IL-17, the severity of *C. muridarum* genital infection and associated oviduct pathology are significantly attenuated, however neither infection or pathology can be reduced further by vaccination protocols that effectively protect WT mice.

## Introduction

The obligate, intracellular human pathogen *Chlamydia trachomatis* is the most common bacterial sexually transmitted disease worldwide and the cause of preventable blindness (trachoma) in developing countries (WHO 2007). More than 100 million new infections occur annually with untreated genital infection causing pelvic inflammatory disease (PID) in women and prostatitis in men, leading to infertility in both sexes. Currently the cost of treating pelvic inflammatory disease (PID) alone in the United States is in excess of US$4 billion annually (WHO 2012). To study the mechanisms underlying immune protection against infection as well as the infection induced inflammation that results in oviduct occlusion, the mouse model of genital *C. muridarum* infection is commonly used as it closely replicates many facets of human *C. trachomatis* infection [1] [2] [3]. Mouse studies have shown that CD4+ T-helper1 (Th1) cells and interferon gamma (IFNγ) dependent immunity is essential for resolution of a primary genital tract infection [2], whilst antibodies and CD8+ T cell-mediated immunity contribute to the host’s resistance to chlamydial reinfection [4] [5] [6].

The classical Th1 and Th2 paradigm, described by Mosman and Coffman [7] has underpinned much immunological research for the past 20 years and has recently been extended to include a number of other CD4 subsets defined by cytokine secretion patterns (reviewed in 8). One of the newly described T helper cell populations Th17 cells are characterized by the secretion of the cytokine interleukin 17 (IL-17) [9] [10] and have been implicated in inflammation [11], autoimmunity [12] and protection against various fungal [13] [14] and viral [15] pathogens. Importantly, IL-17 has also been shown to be important in protection against a number of bacterial pathogens including the extracellular bacterium *Klebsiella pneumoniae* [16] and intracellular bacteria including *Mycobacterium tuberculosis*, *Mycoplasma pneumoniae* and *Listeria monocytogenes* [17] [18] [19]. As *Chlamydia* has a bi-phasic developmental cycle, with both an extracellular and intracellular stage, and because the cytokine milieu necessary to activate Th17 cells in humans and mice is produced in response to a chlamydial genital infection [20], the role of Th17 cells during chlamydial infection is of interest. Subsequent to Th17 cell activation, an increase in IL-17 production at the early stages of infection may benefit the infected host by (i) inducing chemokines (IL8/CXCL8) that recruit neutrophils and (ii) promoting IL-22 mediated production of defensins at the site of infection [21,22]. In contrast, IL-17 may play a role in stimulating the production of the neutrophil enzyme matrix metalloproteinase-9 (MMP-9), which in turn increases neutrophil infiltrates in the upper genital tract and stimulates adverse hydrosalpinx formation via enzymatic modification of chemokines and production of chemotactic collagen peptides [23] [24]. Indeed, IL-17-mediated activation of MMPs has been demonstrated to play a role in a number of inflammatory conditions including inflammation of mouse airways [25], cartilage destruction in mouse models of arthritis [26,27], hepatocellular carcinoma metastasis [28] and inflammation associated with human atherosclerosis [29], a condition that has been linked to *C. pneumoniae* infection [30].

We, and others have shown that immunization of mice via the intranasal (IN) route is an effective means of eliciting protective immunity against genital *C. muridarum* infection [31] [32] [33,34]. Interestingly, recent studies by Zygmunt et al. [35] have shown that the IN route of immunization preferentially induces a Th17 response, further suggesting that IL-17 may have a role in genital tract chlamydial immunity. Lu et al. [36] however, showed that the protection against genital infection afforded by IN immunization with live *Chlamydia* correlated with a T cell response characterised by high IFNγ and low IL-17 production. Furthermore, Yu et al. [37] immunized mice by the subcutaneous route and found that protection against genital *C. muridarum* infection was conferred by multifunctional T cells, secreting either IFNγ and TNFα or IFNγ and IL-17. Thus the data on the role of Th17 cells/IL-17 in protection against genital chlamydial infection is not fully understood.

*C. muridarum* lung infection has been shown to stimulate early IL-17 production after intranasal delivery of the bacterium. Neutralising the initial IL-17 response enhanced *Chlamydia* replication and decreased mouse survival [38], suggesting that IL-17 plays a protective role during a chlamydial lung infection. Interestingly, a prior *C. muridarum* respiratory infection also protects against a subsequent genital infection [39]. In contrast to respiratory tract infection, IL-17 receptor-deficient mice given a primary *C. muridarum* genital tract infection resolved the infection normally in the absence of IL-17 signalling [22]. These conflicting data emphasize that the role of IL-17 in the natural clearance of a chlamydial genital infection, the development of inflammation following infection and in vaccine-induced protective immunity may be multi-faceted and remain to be fully elucidated.

In this study, we have infected IL-17-/- and wild type (WT) mice with *C. muridarum* and monitored the clearance of infection, the development of inflammatory pathology (hydrosalpinx) and vaccine-induced protection against both bacterial burden and pathology following IN and transcutaneous immunization (TCI) to determine the requirement for IL-17 during each of these phases of infection. We found that both the magnitude and duration of *C. muridarum* genital infection and the development of hydrosalpinx were reduced in IL-17-/- mice compared to WT BALB/c mice. This correlated with significantly decreased recruitment of both neutrophils and macrophages into the oviducts of IL-17-/- mice, decreased matrix metalloproteinase activity and increased serum levels of *Chlamydia* -neutralizing antibody. Furthermore, immunization by either the IN [40] or TCI [41] routes reduced both infection and hydrosalpinx in WT mice, which exhibited higher T cell proliferation and IFNγ production following IN immunization, but provided no extra protection against either infection or pathology in IL-17-deficient mice.

## Materials and Methods

### Ethics statement

All procedures were approved by the Queensland University of Technology Animal Research Ethics Committee (Approval 0700000346) and carried out in strict accordance with any recommendations. All efforts were made to minimize suffering.

### *Chlamydia* *stocks* , animals and infection

*Chlamydia muridarum* (Weiss: ATCC VR-123, Virginia, USA) was propagated in McCoy cells and purified using a discontinuous Renografin gradient (Bayer Ltd, Pymble NSW, Australia) to yield purified elementary bodies, which were aliquoted in sucrose-phosphate-glutamate (SPG) and stored at -80 °C. Female BALB/c WT (Animal Resource Centre, Perth, Australia) or IL-17 deficient mice bred on a BALB/c background [42] were used at 6-8 weeks of age. Mice were given 2.5 mg of medroxyprogesterone acetate (Depo-Provera, Pfizer, NSW, Australia) subcutaneously to synchronise the estrous cycle and increase their susceptibility to a chlamydial infection. Seven days later, mice received an intravaginal inoculation of 5x10^4^ inclusion forming units (IFUs) of live *C. muridarum*. Non-infected controls received SPG only. The experiment was repeated three times, with six mice per experimental group and three mice per control group.

### Monitoring of live *Chlamydia* *shedding* from vaginal swabs

In order to characterise the course of the chlamydial infection in WT and IL-17 -/- mice, vaginal swabs (Copan, Murrieta, CA) were taken every 3 days for 21 days. Following collection, each swab was immersed in 500 µl of SPG buffer in a microfuge tube with 3 sterile glass beads. The swab was vortexed and stored at -80 °C. After all samples were collected, the tubes were quickly thawed and again vortexed to release the organisms from the swab. McCoy cell monolayers grown on 48 well plates were inoculated with 10 µL of specimen from swabs collected on days 3 to 9, and 25 µL from specimens collected on days 12 to 21. After 4 h incubation at 37 °C, the inoculum was removed and replaced with fresh media containing 1 µg/mL of cycloheximide. The plate was incubated for a further 24 h then fixed with methanol. The inclusions were visualised by staining with rabbit anti-*C. trachomatis* antibody (Pierce/Progen, Richards, Australia) and the Immunopure ABC/DAB staining kit (Pierce/Progen, Richards, Australia). Ten random fields of view per well were counted per swab sample. The total number of IFU was calculated by averaging the number of counts per field and multiplying by the number of IFU per well and the dilution factor.

### Evaluating chronic pathology

Mice were sacrificed 35 days following the primary infection and the female reproductive tissues were removed. At the macroscopic level, upper genital tract pathology was assessed by measuring the size of hydrosalpinx as previously described [43,44].

### Staining of oviducts and uterine horns for neutrophils and macrophages

Female BALB/c WT (n=3) and IL-17 -/- mice (n=3) were progesterone treated and infected with *C. muridarum* as described above. The animals were euthanized on day 6 and oviducts and uterine horns were collected from each animal. Tissues were fixed in 10% neutral buffered formalin overnight. Immunohistochemistry was performed at the Queensland Institute of Medical Research (QIMR). Samples were paraffin embedded and sections were cut and affixed to adhesive slides, then subjected to antigen retrieval and blocking. For neutrophil detection, primary antibody rat anti-mouse neutrophil Gr-1 (NIMP-R14; Abcam, Sapphire Bioscience, NSW, Australia) was added, followed by Rat Probe (Biocare Medical, CA, USA) and Rat-on-Mouse HRP-Polymer (Biocare Medical), and then developed with Betazoid DAB (Biocare Medical). For macrophage detection, primary antibody rat anti-mouse F4/80 (CI:A3-1; Abcam) was added, followed by alkaline phosphatase conjugated goat anti-rat Ig (Chemicon, CA, USA), then developed with Vulcan Fast Red Chromogen (Biocare Medical). Slides were counterstained in Mayer’s haematoxylin, scanned at QIMR using Aperio Scanscope XT and analyzed using Aperio ImageScope software v12. Staining intensity was quantified by measuring strong positive pixels using preconfigured positive pixel count v9 algorithm and expressing as a percentage of total pixels per tissue.

### Evaluation of metalloproteinase activity

Cervix, uterine horn and oviduct tissues were collected on days 7, 14 and 21 post-infection (p.i.) into 500 µL of phosphate buffer saline (PBS) and stored at -80 °C. For assay of metalloproteinase activity, the tissues were homogenized and processed for gelatin zymography, as described previously [45]. In addition, tissue homogenates were used for the antigen capture enzyme-linked immunoassay detection of total MMP-2 and proMMP-9, according to the manufacturers instructions (R&D Systems, Inc., Minneapolis, MN). Gelatin zymography provides relative assessment of gelatinase activity while ELISA provides assessment of zymogen MMP-9 present in the targeted tissue.

### Antigen and adjuvants

Major Outer Membrane Protein (MOMP, MBP-tagged) was prepared, as previously described [41], from transformed *Escherichia coli* (DH5α [pMMM3]) containing the recombinant MBP-MOMP encoding pMAL-c2 vector. Synthetic CpG-1826 oligonucleotides, with a phosphorothioate backbone (5’-TCCATGACGTTCCTGACGTT- 3’, Sigma-Aldrich, Castle Hill, NSW, Australia), and cholera toxin (CT, Sapphire Bioscience, Waterloo, NSW, Australia) adjuvants, were prepared in sterile PBS.

### Intranasal (IN) Immunization

Mice (n=5 per group) were lightly sedated with 2-4% Isoflurane in 4 L/min oxygen. Vaccine containing 100 µg MBP-MOMP, 5 µg CpG-1826 and 2.5 µg CT was delivered intranasally in a 10 µL volume via micropipette, with each nare receiving 5 µL. Immunizations were administered 3 times at weekly intervals, and an immunization booster was given 2 weeks following the third immunization.

### Transcutaneous immunization

Mice (n=5 per group) were anaesthetised intraperitoneally with xylazine (90 mg/kg) and ketamine (10 mg/kg). A small area (^≈^1 cm^2^) of the lumbar region was shaved and swabbed with acetone. The area was then swabbed with a skin permeabilising solution containing PBS and 100% ethanol in equal volumes, with 0.33% w/v 1-Dodecylpyridinium chloride (DPC), 0.33% w/v Isopropyl myristate (IPM) and 0.33% w/v N-methylpyrrolidone (NMP). Chemical residue was removed by swabbing the exposed skin with sterile PBS. Before administering vaccine, 12.5 ng of recombinant-mouse GM-CSF (Invitrogen, Mount Waverley, VIC, Australia) was added to the shaved region. Vaccine containing 100 µg MBP-MOMP, 5 µg CpG-1826 and 2.5 µg CT was then delivered in a 50 µL volume to the immunization site and allowed to absorb into the skin. The site was covered with gauze and Flexifix, which was held in place with surgical tape. This was removed 24 h post immunization, and site was swabbed with sterile PBS to remove any remaining vaccine. Immunizations were administered three times at weekly intervals, and an immunization booster was given 2 weeks following the third immunization.

### Live infection control (LIC)

Mice (n=5 per group) were given 2.5 mg medroxyprogesterone acetate subcutaneously and infected intravaginally 7 days later with 5x10^4^ IFU of live *C. muridarum* as described. Mice were re-infected 42 days after the primary infection.

### Sample collection

Vaginal washes were collected by flushing the vaginal vault with 40 µL sterile PBS. This was repeated daily for 4 days, concluding on day of euthanasia. Vaginal washes collected across the timepoints were combined, so that each animal was sampled across an entire estrus cycle. Mice were euthanized on day 35 through intraperitoneal injection with 1 mL/2 kg sodium pentobarbitone. Blood was collected via intracardiac puncture and then centrifuged at 12 000 x g for 45 minutes to obtain serum. Both serum and vaginal washes were stored at -20 °C. Spleens were surgically removed and pooled for each group.

### ELISA analysis

ELISA 96-well plates (Interpath, Heidelberg West, VIC, Australia) were coated with 2 µg MBP-MOMP/well in borate buffer solution (BBS), then incubated at 4 °C overnight. Plates were washed 3x with PBS containing 0.5% Tween 20 (PBS-T) and blocked with 5% fetal bovine serum in PBS-T at 37 °C for 2 h. ELISAs were performed with serum and vaginal wash samples as previously described [34,41]. MBP-MOMP specific IgG and IgA were detected with 1/1000 HRP- conjugated goat anti-mouse IgG and goat anti-mouse IgA respectively (Southern Biotech, InVitro Technologies, VIC, Australia). Samples collected from naïve mice were used as negative controls to determine the end point titre (EPT) threshold, which is the mean absorbance of the negative control + 2 standard deviations. EPT of the test samples were calculated as the inverse of the sample dilution at which absorbance values were equivalent to the EPT threshold.

### In vitro neutralization of *C. muridarum* infection

Sera collected from mice post immunization and 35 days post-infection (LIC) was diluted 1/10, 1/20, 1/40, and 1/80 in media in 96 well round bottom microplates (Nunc). Each well was incubated with 1.25 x 10^4^ IFU of *C. muridarum* for 1 h at 37 °C. Following incubation, the contents were transferred from each well to a corresponding well in 96 well flat bottom microplate (Nunc, Thermo Scientific, Australia)) containing 80% confluent McCoy cell monolayers. The plates were incubated at 37 °C and infection proceeded for 24 h as described above. Following fixation, wells were blocked with 5% fetal bovine serum in PBS-T at 37 °C for 2 h. Wells were incubated with 1/500 sheep anti-*C. muridarum* MOMP for 1 h at 37 °C, then with 1/2000 donkey anti-sheep IgG Alexa Fluor 488 (Invitrogen) and 1/40 000 DAPI nucleic acid stain (Invitrogen) to visualise inclusions and cells respectively. These were photographed using fluorescence microscopy on x4 magnification and percent infectivity was determined using MetaMorph counting software (MDS Analytical Technologies). Neutralisation was calculated by comparing the change in percent infectivity of a sample well, to a control well containing no sample.

### Splenocyte proliferation

Spleens were processed as previously described [41]. Splenocytes were added in quadruplets to 96-well plates at 5x10^5^ cells/well, then stimulated with 10 µg MBP-MOMP/well and incubated at 37 °C for 3 days. Half the media from each well was then removed and stored at -80 °C for cytokine assessment; [methyl-^3^H] Thymidine (MP Biomedicals, Australasia, Seven Hills, NSW, Australia) was then added to the remaining media at 0.5 µCi/well. The plates were incubated at 37 °C overnight, then harvested onto a filtermat using a Brandel 96-well plate harvester. Filtermats were wetted with Betaplate Scint (PerkinElmer) and the incorporated radioactivity was measured using a 1450 MicroBeta Liquid Scintillation and Luminescence Counter plate reader (PerkinElmer).

### Cytokine assessment

Supernatants from stimulated splenocytes that were collected for cytokine assessment were centrifuged at 500 x g for 10 mins to remove debris. Quadruplet supernatants were pooled and analysed as duplicates on Bio-Plex Pro mouse cytokine immunoassay for IL-2, IL-4, IL-5, IL-10, IL-12(p70), GM-CSF, IFNγ and TNFα (Bio-Rad, Regents Park, NSW, Australia), according to the manufacturers instructions.

### Statistics

All statistical analyses were performed using GraphPad Prism v.5. Statistical significance was determined using unpaired, two-tailed Student’s t-test with 95% confidence interval (C.I.). Statistical significance was accepted at p <0.05.

## Results

### Characterization of the course of a chlamydial infection in wild type (WT) versus IL-17 knockout mice

**Figure 1:**
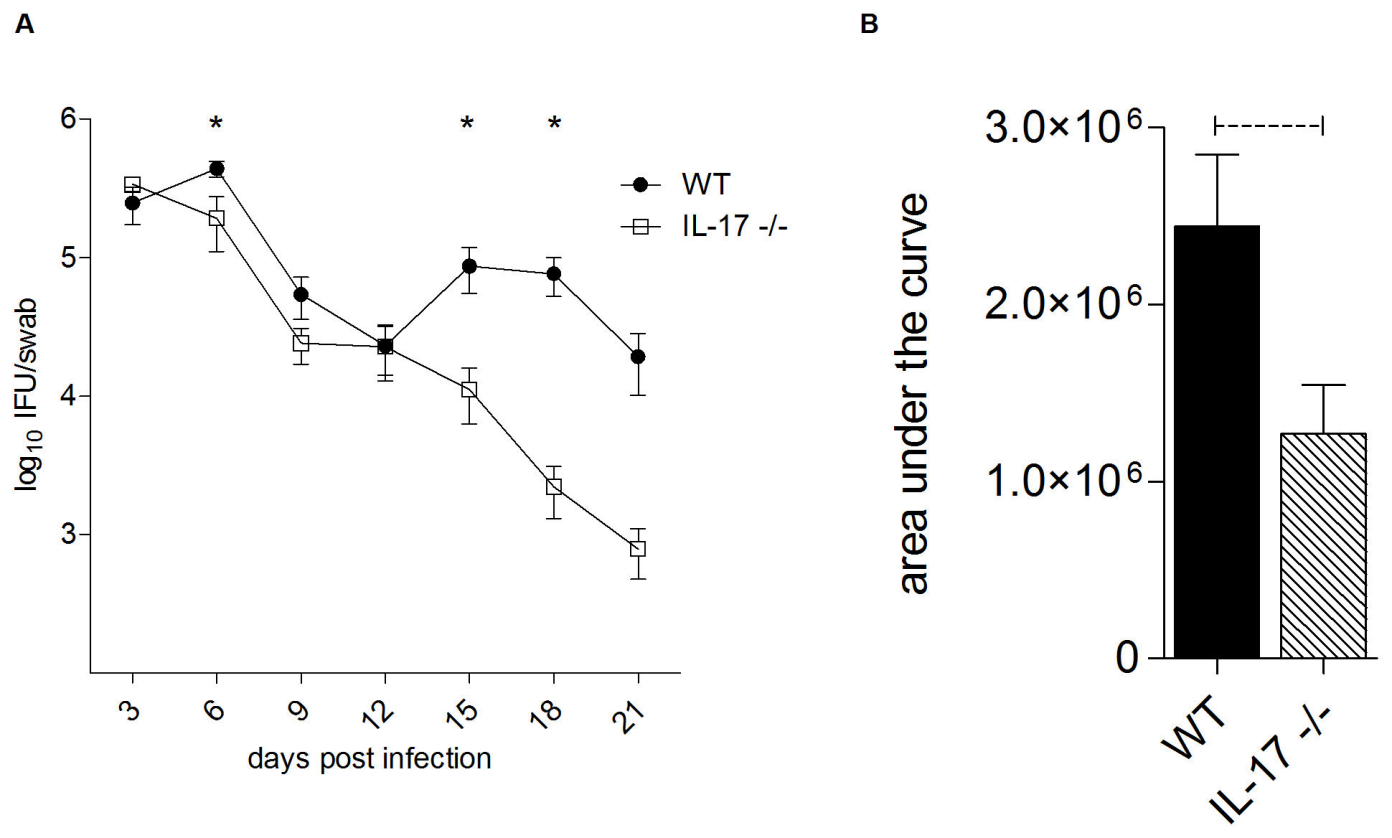
Clearance of vaginal *C. muridarum* infection in BALB/c and IL-17-/- mice. Vaginal swabs were collected every 3 days following challenge of progesterone primed mice with 5 x 10^4^ *C. muridarum*. The number of IFU per swab was determined by culture on McCoy cells. The numbers of IFU per swab (A) and the area under the clearance curves (B) are presented. (*) or dotted bar represents p<0.05 significance.

Both WT and IL-17-/- mice were infected intravaginally with *C. muridarum*. Swabs were collected every 3 days for 21 days for evaluation of chlamydial shedding and infectious burden. As shown in Figure 1A, the absence of IL-17 had little effect on bacterial clearance from the lower genital tract between days 3 to 12 although vaginal shedding was significantly reduced at day 6 p.i. Furthermore, *C. muridarum ompA* transcripts were 2-5-fold higher in oviducts of WT mice compared to IL-17-/- mice at day 6 p.i. (data not shown). However, the chlamydial burden in the lower genital tract was significantly higher in WT mice compared to the IL-17-/- mice during the later stage of infection at days 15 and 18 p.i. (p<0.05). At day 21, all WT mice were still shedding *C. muridarum* into the vaginal vault, whereas 33% of IL-17-/- mice had cleared the infection (data not shown). Calculation of the area under the curve (AUC) allows a combined comparison of both the magnitude and duration of infection. AUC analysis (Figure 1B) confirms the reduced infection in IL-17-/- mice. These data show that in the absence of IL-17, mice clear a vaginal *C. muridarum* infection more quickly than their WT counterparts and the overall burden of *Chlamydia* is reduced.

### Infection induced chronic disease in the upper genital tract

**Figure 2:**
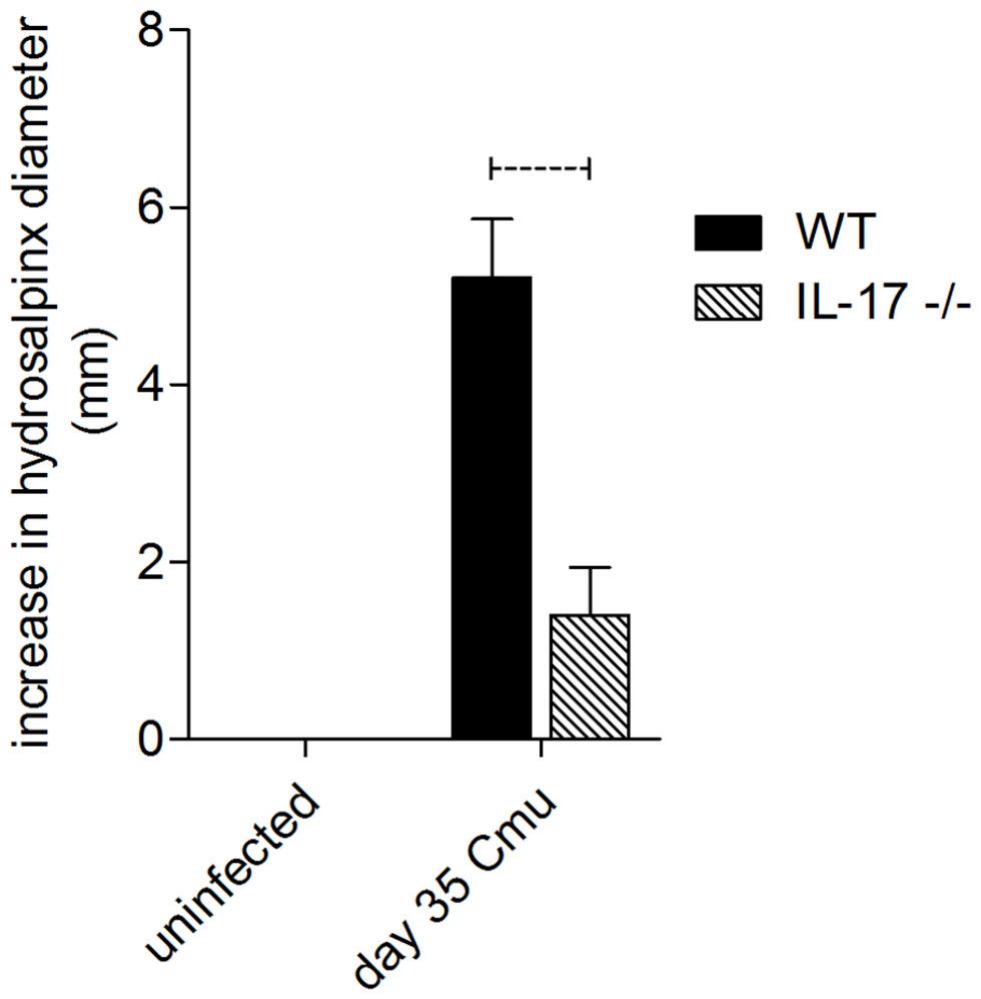
Infection-induced occlusion of oviducts. Hydrosalpinx was measured at 35 days post infection following challenge of WT BALB/c and IL-17-/- mice with 5 x 10^4^ *C. muridarum*. Dotted bar represents p<0.05 significance.

**Figure 3:**
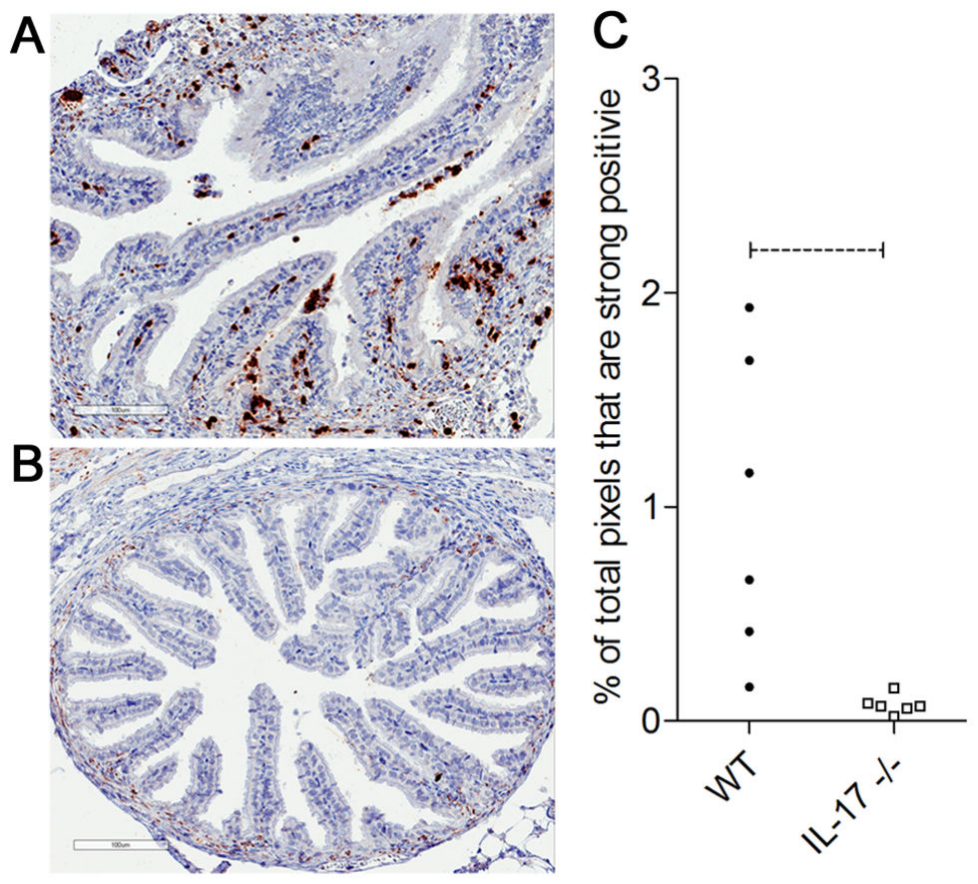
Neutrophil infiltration of oviducts following *C. muridarum* infection. Oviducts were collected from WT BALB/c mice (A), IL-17-/- mice (B) 6 days post infection and stained for Ly-6G/Ly-6C as described in materials and methods. Staining of oviduct tissues was quantitated by image analysis (C) as described in methods. Scale bar = 100 µM. Dotted bar represents p<0.05 significance.

**Figure 4:**
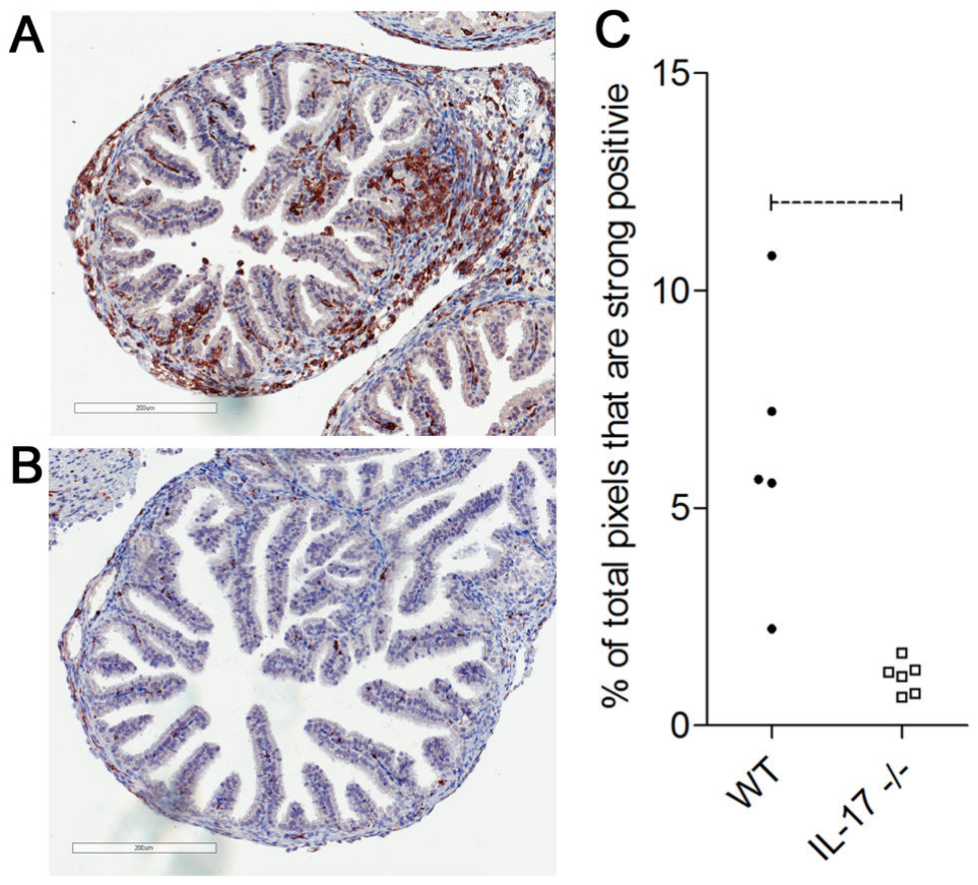
Macrophage infiltration of oviducts following *C. muridarum* infection. Oviducts were collected from WT BALB/c mice (A), IL-17-/- mice (B) 6 days post infection and stained for F4/80 as described in materials and methods. Staining of oviduct tissues was quantitated by image analysis (C) as described in methods. Scale bar = 200 µM. Dotted bar represents p<0.05 significance.

*C. muridarum* infection of WT mice caused the development of significant hydrosalpinx compared to non-infected WT controls. However, infected IL-17-/- mice did not develop significant hydrosalpinx and levels of inflammation were no different to that seen in non-infected WT or IL-17-/- mice (Figure 2). The lack of gross disease pathology in IL-17-/- mice was supported by the histological analysis of innate immune cell infiltration into the oviducts. At day 6 p.i., the oviducts of WT mice contained significant numbers of Gr-1+ neutrophils (Figure 3A) whilst the oviducts of IL-17-/- mice contained very few infiltrating neutrophils (Figure 3B). Image analysis of Gr-1+ cell numbers in the oviducts at day 6 p.i. confirmed that significantly more neutrophils were recruited into the oviducts of WT mice compared to IL-17-/- mice (Figure 3C). A similar observation was seen with oviduct sections stained with F4/80, revealing infiltrating macrophages. Oviduct macrophage numbers in WT mice (Figure 4A) were greatly increased at day 6 p.i. compared to oviducts of IL-17-/- mice (Figure 4B) and the increased macrophage numbers in WT mice were significantly higher (Figure 4C) at this time.

### Infection-induced MMP2 and MMP9 expression in reproductive tract tissues

**Figure 5:**
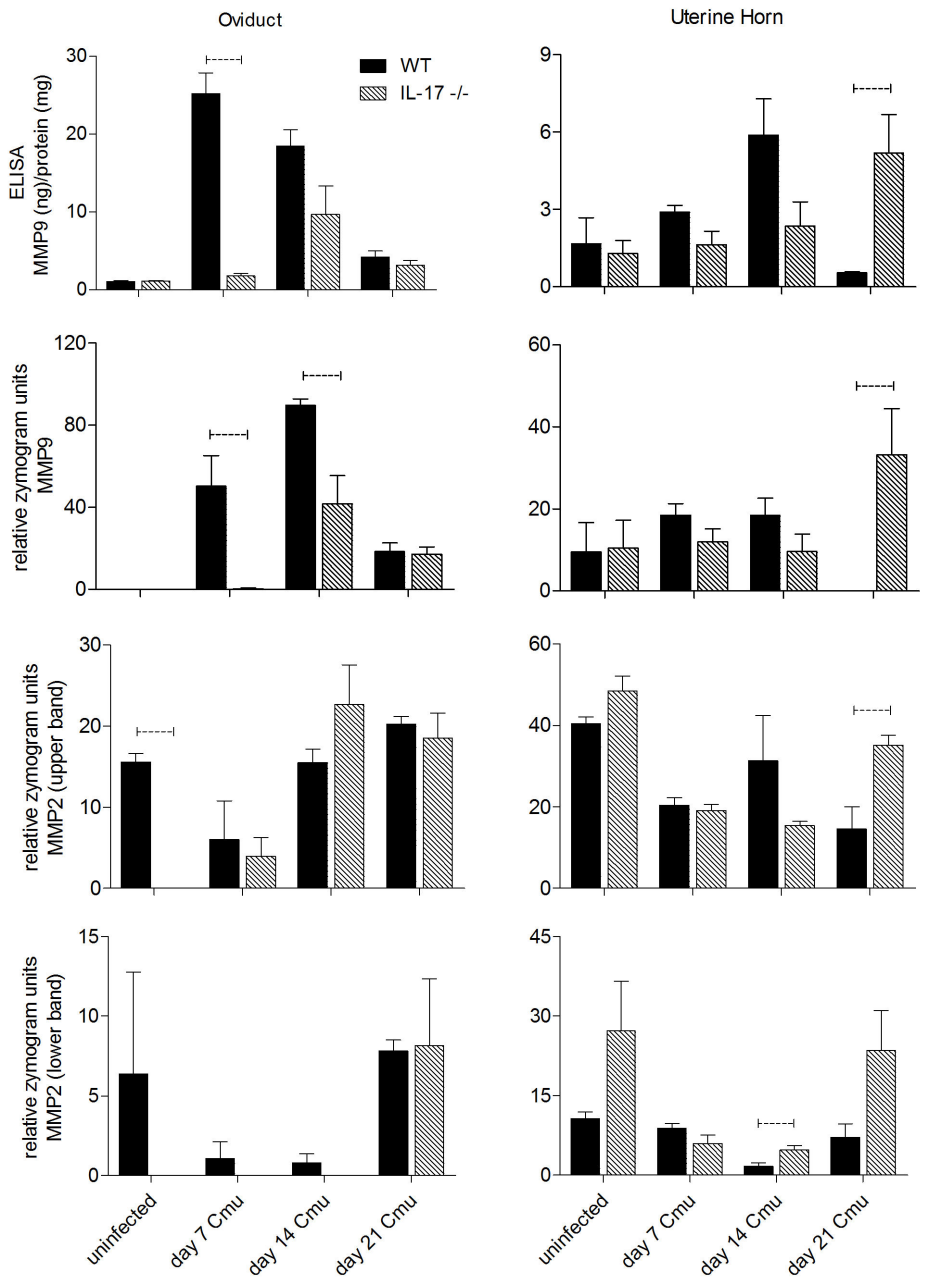
Expression of matrix metalloproteinase MMP9 and MMP2 in oviducts and uterine horns of wild type BALB/c and IL-17-/- mice 7 days post infection with *C. muridarum.* MMP expression was determined by both ELISA and gelatin zymography as described in materials and methods. Dotted bar represents p<0.05 significance.

Because IL-17 has been associated with the activation of MMPs in other inflammatory conditions, we investigated the expression of MMP2 and MMP9 in reproductive tract tissues of WT and IL-17-/- mice following *C. muridarum* infection. Oviducts and uterine horns were collected at day 7, which represents the peak of infection following *C. muridarum* challenge. Pro-MMP9 levels were significantly increased in oviducts from WT mice compared to IL-17-/- mice at 7 days p.i. as determined by both ELISA (p<0.0005, indicating zymogen MMP-9) and gelatin zymography (p<0.05, indicating active MMP-9, Figure 5) and remained increased at day 14 p.i., although this was only significant as determined by zymography (p<0.05). Late in the infection (day 21), the trend was reversed and higher levels of pro-MMP9 were found in the uterine horns of IL-17-/- mice compared to WT mice (p<0.05, Figure 5). Pro-MMP2 was higher in oviducts of uninfected WT mice compared to IL-17-/- mice (p<0.0005), however there was no difference between levels in infected WT and IL-17-/- mice at any time p.i. Higher levels of pro-MMP2 were found in uterine horns of IL-17-/- mice compared to WT at 21 days p.i. (p<0.05) and active MMP2 was also increased in IL-17-/- mice at 14 days p.i. (p<0.05).

### Vaccine-induced serum and vaginal MOMP antibodies are increased in IL-17-/- mice

**Figure 6:**
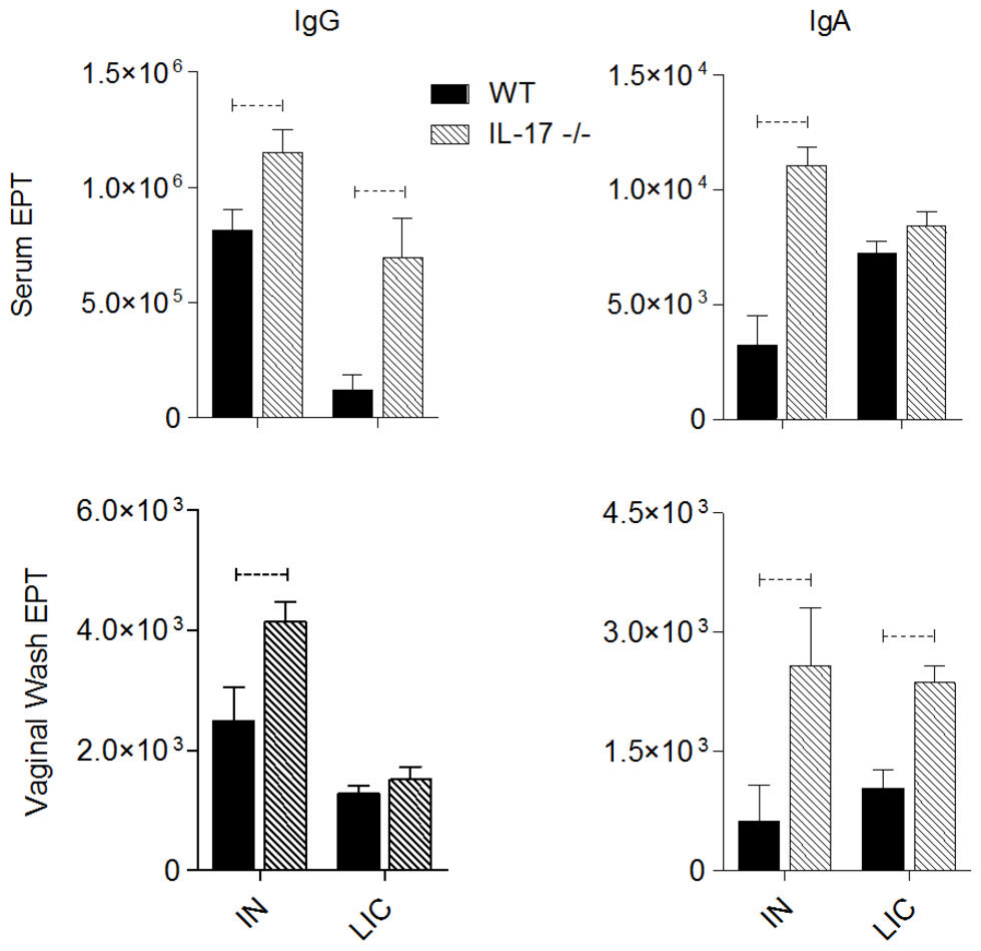
MOMP-specific IgG and IgA in serum and vaginal wash fluids following intranasal (IN) immunization and resolution of a primary *C. muridarum* infection (LIC). MOMP-specific IgG and IgA antibodies were measured by ELISA as described in materials and methods following intranasal immunization or resolution of a primary vaginal infection. Dotted bar represents p<0.05 significance.

We have previously shown that IN immunization protects against *C. muridarum* genital tract infection [31,32]. Furthermore, Zygmunt et al. [35] have shown that IN immunization preferentially induces Th17 cell responses, regardless of the adjuvant used. It was therefore of interest to determine if IN immunization of IL-17-/- mice could protect against infection and oviduct inflammation. Serum and vaginal washes were collected following IN immunization with MOMP and CT/CpG and MOMP-specific IgG and IgA levels determined by ELISA (Figure 6). Anti-MOMP antibody responses elicited by a natural infection were also determined. MOMP-specific IgG and IgA in serum was significantly higher in IL-17-/- mice compared to WT mice (p<0.05) following IN immunization and anti-MOMP IgG levels elicited by a natural infection were also significantly higher in the IL-17-/- animals (p<0.05, Figure 6). The IgG subclass of the vaccine-induced serum IgG response was also significantly different in IL-17-/- mice. IN immunization of WT mice resulted in an IgG response dominated by the IgG1 subclass (IgG1: IgG2a ratio 26.2:1) whereas in the serum of immunized IL-17-/- mice the IgG1 response was less dominant (IgG1: IgG2a ratio 3.6:1). Serum IgA levels following natural infection were equivalent between strains whereas IN immunization elicited higher serum IgA levels in IL-17-/- mice compared to WT (p< 0.05, Figure 6). MOMP-specific IgG levels were significantly higher in vaginal washes of IL-17-/- mice compared to WT (p<0.05) following immunization but levels elicited by natural infection were equivalent (Figure 6). Significantly higher levels of both vaccine-induced and infection-induced IgA in VL were found in IL-17-/- mice compared to WT controls (p<0.05).

### In vitro neutralization of *C. muridarum* by serum from WT and IL-17-/- mice

Despite the increased levels of serum antibodies in IL-17-/- mice following IN immunization the neutralizing activity of serum from IL-17-/- mice was not significantly greater than that of serum from similarly immunized WT mice (Table 1). At a 1:10 dilution, serum from knockout mice neutralized infection by 64 + 8% whilst serum from WT mice reduced infection by 59 + 6%. Although the *Chlamydia* -neutralizing capacity of sera from WT and IL-17-/- mice following IN immunization was equivalent this was not the case for sera collected following resolution of a natural infection (LIC). Sera from IL-17-/- mice, collected 35 days post-infection had significantly greater neutralizing activity than sera collected from WT mice. WT serum, at a 1:20 dilution failed to neutralize in vitro *C. muridarum* infection whilst sera from IL-17-/- mice still neutralized infection by ~50% at this dilution.

**Table 1:** Percentage neutralization of *C. muridarum* by diluted serum collected from IN immunised and LIC wild type and IL-17 - /- mice.

**IN**							**LIC**						
	WILD TYPE			IL-17 -/-				WILD TYPE			IL-17 -/-		
1/10	58.7	±	5.5	64.0	±	8.4	1/10	47.5	±	14.9	63.1	±	10.7
1/20	26.7	±	11.3	36.9	±	10.5	1/20		-		50.4	±	25.8
1/40	14.7	±	8.9	5.7	±	9.9	1/40		-			-	
1/80		-			-		1/80		-			-	

Values are mean +/- SD (n=5).Vaccine-induced T cell proliferation and cytokine production in IL-17-/- and WT mice

**Figure 7:**
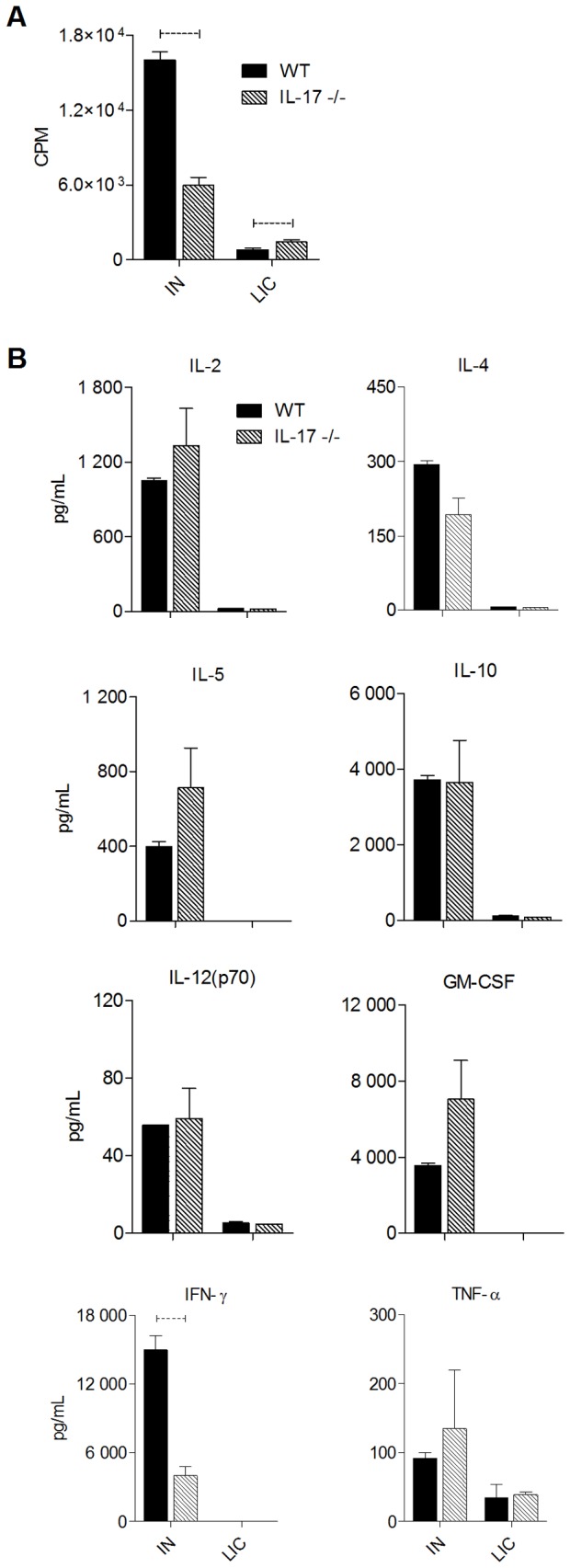
MOMP-induced splenocyte proliferation and cytokine secretion following intranasal (IN) immunization or following resolution of infection (LIC). Splenocytes were stimulated *in* *vitro* with MOMP and proliferation (A) determined by ^3^H-thymidine incorporation (CPM = counts per minute). Cytokines secreted by MOMP-stimulated cells (B) were measured by Bio-Plex assay as described in methods. Dotted bar represents p<0.05 significance.

MOMP-induced *in vitro* splenocyte proliferation was significantly higher in IN immunized WT mice compared to IL-17-/- mice (p<0.05, Figure 7A). Interestingly, proliferation of splenocytes isolated from animals that had recovered from a natural infection, although lower overall than proliferation levels in immunized animals, was higher in the IL-17-/- mice (p<0.05). Supernatants from *in vitro* MOMP-stimulated spleen cells were also assayed for cytokine levels by Bio-Plex assay (Figure 6B). Of all the cytokines analysed, only IFNγ levels were different between strains, with levels significantly higher in supernatants from stimulated WT cells compared to IL-17-/- cells (p<0.05, Figure 7B). In both WT and IL-17-/- mice that had recovered from a natural infection (LIC), only low levels of TNFα and IL-12 were produced following *in vitro* re-stimulation of splenocytes with MOMP. This suggests that during a natural infection MOMP is a major target for humoral immunity (Figure 6) but is not a dominant T cell antigen.

### IN immunization reduces infection in WT but not IL-17-/- mice

**Figure 8:**
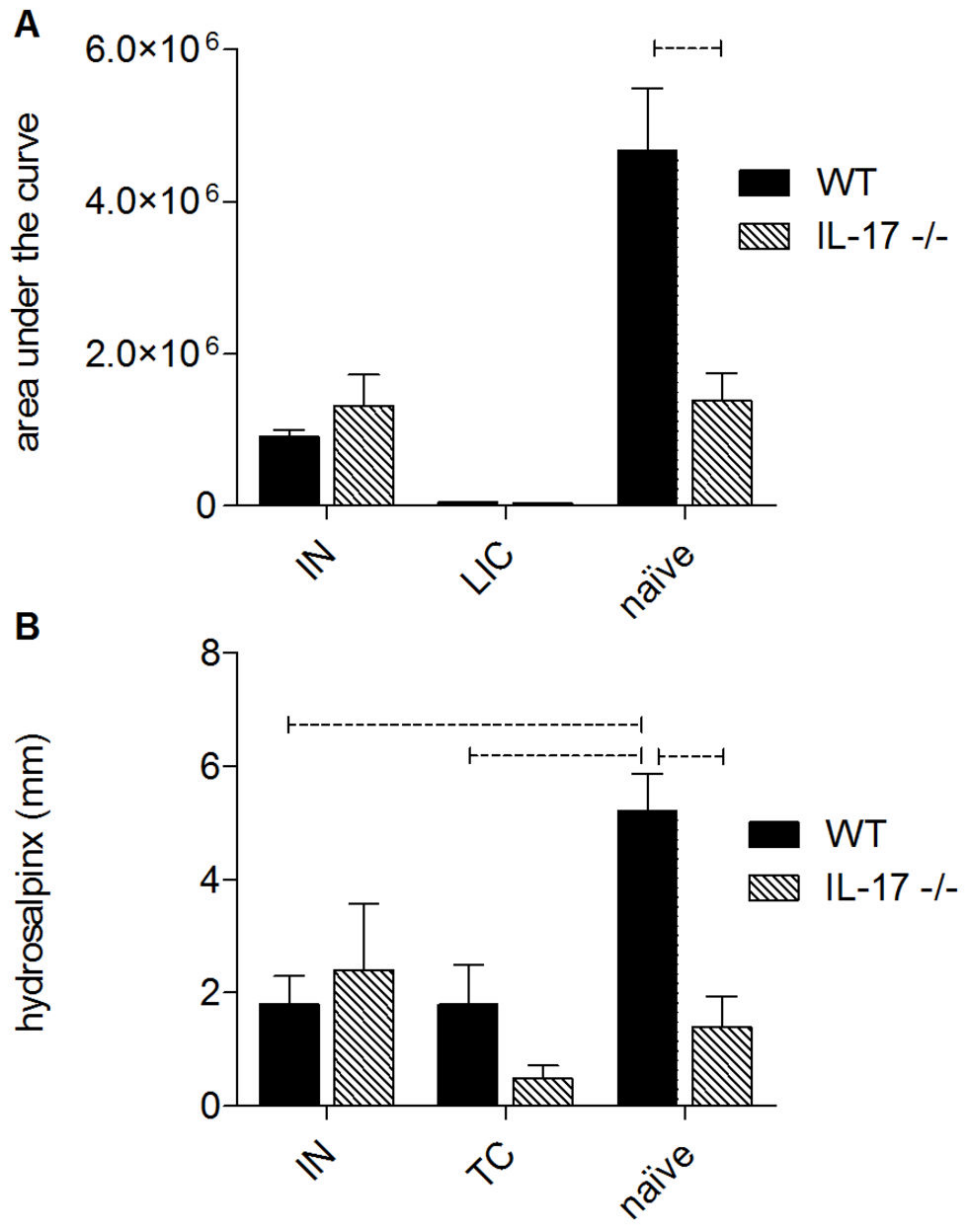
The effect of immunization or a prior infection on chlamydial clearance and development of hydrosalpinx. BALB/c or IL-17-/- mice were immunized by either the intranasal (IN) or transcutaneous (TC) routes or allowed to recover from a *C. muridarum* infection (LIC). Vaginal swabs were collected every 3 days following challenge. The number of IFU per swab was determined by culture on McCoy cells and plotted against time. From these plots, the area under the curve was calculated (A). Hydrosalpinx was measured at day 35 post-infection (B) as described in materials and methods. Dotted bar represents p<0.05 significance.

Following IN immunization, both WT and IL-17-/- mice were challenged intravaginally with *C. muridarum*. WT and IL-17-/- mice that had recovered from a previous *C. muridarum* infection were also included as a prior infection protects against subsequent challenge (Figure 8A, live infection control [LIC]). The bacterial load in swabs collected every 3 days was determined and graphed against time to generate clearance curves (as for Figure 1A). These data are reported as area under the curve (AUC) (Figure 8A), which reflects both the magnitude of shedding at each time point and the duration of infection. Consistent with the data in Figure 1, the AUC in naïve animals was significantly reduced in IL-17-/- mice compared to WT (p<0.05, Figure 8A), reflecting both a shorter duration of infection and a lower burden. IN immunization of WT mice significantly reduced the AUC as expected (p<0.05 compared to non-immunized WT mice, Figure 8A), however immunization of IL-17-/- mice did not provide further protection as the AUC for immunized IL-17-/- mice was not significantly different from that of naïve knockout mice (Figure 8A). Interestingly, a prior genital tract infection was able to protect both WT and IL-17-/- mice against subsequent re-infection (Figure 8A, LIC) as recoverable chlamydial IFU were reduced to below the limit of detection in both strains. Protection against the development of inflammatory pathology that can cause tubal occlusion has been suggested to be an important requirement for a chlamydial vaccine [46]. We therefore determined if vaccination of WT and IL-17-/- mice prevented the development of oviduct pathology (Figure 8B). Consistent with the data in Figure 2, *C. muridarum* infection of naïve mice induced severe hydrosalpinx in WT but not IL-17-/- mice. IN immunization of WT mice protected against the development of hydrosalpinx (P<0.001 compared to non-immunized WT) but immunization of IL-17-/- mice did not further reduce inflammation below that seen in infected naïve knockout mice (p>0.05, Figure 8B). IN immunization is known to preferentially elicit Th17 responses [35], therefore, we also investigated if TC immunization could induce protection against hydrosalpinx in IL-17-/- mice as we had shown previously that TC immunization protected BALB/c mice against genital tract chlamydial infection [41,47]. TC immunization of WT mice with MOMP and CT/CpG reduced the bacterial burden (AUC) in WT mice by around 20% (data not shown) and significantly reduced the severity of hydrosalpinx compared to non-immunized WT mice (p<0.0025, Figure 8B). However, TCI of IL-17-/- mice did not reduce either the infectious burden (AUC, data not shown) or the degree of hydrosalpinx compared to naïve IL-17-/- mice (Figure 8B).

## Discussion

In this study, we show that the magnitude and duration of a vaginal *C. muridarum* infection in IL-17-/- mice is significantly reduced compared to WT mice, with the reduction in infection most evident late (days 12-21) in the course of infection (Figure 1A). Furthermore, following resolution of infection, IL-17-/- mice fail to develop the severe hydrosalpinx that is seen in infected WT mice at day 35 p.i. (Figure 2). All infected WT mice developed severe unilateral, or in most cases bilateral hydrosalpinx while oviducts isolated from IL-17-/- mice were no different to those in uninfected WT or knockout controls. Infiltration of neutrophils (Figure 3) and macrophages (Figure 4) into the oviducts at the peak of infection (day 6), was significantly reduced in IL-17-/- mice. Pro MMP9 levels, as measured by ELISA, were increased in oviducts of WT mice compared to IL-17-/- mice at day 7 p.i. and remained elevated at day 14 p.i. and zymography indicated comparatively elevated gelatinase activity in WT mice (Figure 5). During the later stages of infection (day 21) higher pro-MMP9 levels were found in uterine horns of IL-17-/- mice. This delay in elaboration of pro MMP-9 and its related gelatinase activity is likely due to the arrival of other inflammatory cells capable of producing MMP-9, namely macrophages, at the later stage of infection. However, a conclusive *in situ* study of cell types and MMP expression would need to be conducted to be certain of this assertion. Higher levels of pro-MMP2 and active MMP 2 were seen in the uterine horns of IL-17-/- mice (Figure 5). Interestingly, this enzyme is more constitutively expressed in most tissues and is likely involved in tissue remodelling and repair following an inflammatory insult. Taken together, these results indicate that IL-17 either directly or indirectly influences MMP production and activity during infection in this model. Because MMPs have previously proven to be endline effectors in this model, our data provide a link between a single signaling molecule (IL-17) and an endline effector of tissue damage in chlamydial infection. IL-17-induced increases in MMP activity have been implicated in a number of other inflammatory conditions including experimental airways inflammation [25], periodontal disease [48], arthritis [26,27], atherosclerosis [29] and also cancer metastasis [28].

Both the enhanced clearance of infection and the reduced oviduct pathology are likely due to the reduced inflammatory cell influx observed in the IL-17-/- mice compared to WT mice. Macrophages and PMN are major sources of IL-1β, a cytokine that has been implicated in both pathology and clearance of infection [49] [50]. Gr1+ granulocytes in upper genital tract tissues are the major source of MMP9 [51], which was increased in oviducts of WT mice early in infection. Due to the reduced influx of inflammatory cells in IL-17-/- mice, decreased production of these key inflammatory mediators is the most likely explanation for the reduced hydrosalpinx. Infection of neutrophils with *C. muridarum* delays neutrophil apoptotic death [52], potentially providing a source of *Chlamydia* that may reinfect genital epithelial cells. Apoptotic neutrophils infected with *C. pneumoniae* are phagocytosed by macrophages [53] [54], which become infected and can serve as a Trojan horse to spread infection, although this has not been demonstrated for *C* *. muridraum* . However, we and others [55] have shown that macrophages can be directly infected with *C. muridarum* providing a potential reservoir of *Chlamydia* for reinfection. As observed in Figure 1A, there was a second peak of infection at day 15, which could potentially be due to *C. muridarum* released from neutrophils and/or macrophages, which are recruited into the oviducts of WT but not IL-17-/- mice.

Following IN immunization, MOMP-specific IgG and IgA titers were higher in serum and vaginal washes of IL-17-/- mice (Figure 6). IgG1/IgG2a ratios were also different in serum of WT and IL-17-/- mice following IN immunization, showing a Th2-dominant response in WT mice, consistent with the Th2 bias of BALB/c mice, but a more balanced Th_1/2_ response in the knockout mice. Th17 cells have been shown to increase B cell proliferation *in vitro*, to induce isotype switching to IgG1, IgG2a, IgG2b and IgG3 both *in vitro* and *in vivo* following subcutaneous immunization of mice with Freund’s incomplete adjuvant and to enhance germinal centre formation [56]. However, following IN immunization with MOMP plus CT/CpG adjuvants there was no difference in the ability of serum from either group to neutralize *C. muridarum* infection *in vitro* (Table 1). Natural genital infection also induced higher serum IgG and vaginal IgA levels in IL-17-/- mice compared to WT mice and importantly, sera from IL-17-/- mice showed significantly greater neutralizing activity than WT sera (Table 1). This higher neutralizing activity likely contributed to the enhanced resolution of infection in IL-17-/- mice during the late stages of infection. The higher levels of IgA in both serum and vaginal washes of IL-17-/- mice is potentially at odds with the findings of Hirota et al. [57], who found that serum and fecal IgA elicited by oral immunization with cholera toxin was dependent on Th17 cells. Because the development of intestinal Th17 cells is dependent on the presence of commensal microbiota [58], the IL-17 dependence of IgA production may be unique to the intestine and not a feature of IgA production in the genital tract, where the microbial burden is greatly reduced, or IgA responses elicited by nasal immunization, as demonstrated in our study.

Following IN immunization, splenic T cell proliferation after *in vitro* culture with MOMP was significantly greater in WT mice (Figure 7A). Stimulated WT cells also secreted significantly higher levels of IFNγ while levels of the other cytokines assayed were equivalent (Figure 7B). As expected, IN immunization of WT mice significantly reduced both infection (Figure 8A) and inflammatory disease (Figure 8B), however immunization was ineffective in further reducing either infection (Figure 8A) or disease (Figure 8B) in IL-17-deficient mice, most likely due to decreased IFNγ production and the ability to mount a protective Th1 response. Interestingly however, a prior *C. muridarum* infection was able to reduce the magnitude and duration of infection in both WT and IL-17-/- mice (Figure 8A). Collectively our data suggest that IL-17 plays a major role in the development of pathology/disease following chlamydial infection and furthermore, delays infection resolution in BALB/c mice. The cytokine data (Figure 7B) suggest that in the absence of IL-17 the knockout mice were unable to mount an effective Th1 response, as evidenced by the greatly reduced secretion of IFNγ. IL-17 has been shown to be essential for the generation of Th1 responses in murine models of colitis [59] and generation of anti-mycobacterial immunity elicited by immunization with BCG [60]. The mechanisms involve either (i) IL-23/IL-17 dependent suppression of IL-10 [60], facilitating the development of Th1 cells (ii) co-expression of both IL-17 and IFNγ or (iii) the direct conversion of Th17 cells into Th1 cells, indicating the great plasticity of the Th17 cell lineage [61] [62]. Indeed, recent studies have demonstrated a dependence on early IL-17 production, possibly produced by γδ T cells, for generation of effective Th1 immunity against pulmonary Mycobacterial infection elicited by BCG [63] [60], suggesting that IL-17 may be essential for generation of Th1 immunity against intracellular pathogens.

Our findings are in contrast to those of Scurlock et al. [22], who found no difference between the rate of resolution of genital infection and pathology development in WT and IL-17 receptor (IL-17ra-/-) knockout mice. It is possible that deletion of the IL-17ra does not totally abolish IL-17A/F-mediated signalling. Both IL-17A and IL-17F signal through a receptor complex of IL-17ra and IL-17rc (reviewed in 64), two of the five receptors that comprise the IL-17R family. Whilst the current view is that IL-17rc cannot induce signalling in the absence of IL-17ra [64] there are known to be many splice isoforms of IL-17rc and the tissue expression pattern of IL-17rc is different to that of IL-17ra, being low in hematopoietic cells but higher in non-immune cells [65]. Thus it may be possible for some splice variants of IL-17rc alone or complexed with other IL-17R family members to mediate some IL-17 signalling. If this were shown to be the case in the reproductive tract this could provide an explanation for the different outcomes of *C. muridarum* infection in IL-17ra-/- [22] and IL-17-/- mice.

Scurlock et al. [22] also found increased cervical macrophages in IL-17ra-/- mice compared to WT mice following infection and suggested that this may be a compensatory mechanism for reduced Th1 immunity. However, in our study, macrophage numbers in oviducts of IL-17-/- mice were reduced compared to WT (Figure 4C) but infection resolved more quickly and pathology was greatly reduced. IL-17 has recently been shown to favour the activation of pro-inflammatory M1 macrophages over anti-inflammatory M2 macrophages [66]. If the residual macrophages in IL-17-/- mouse tissues were M2 type macrophages this could also have contributed to the reduced hydrosalpinx seen in knockout mice. Preliminary PCR analysis of markers associated with M1 versus M2 development demonstrated increased mRNA levels of M2-associated markers CD206 and FIZZ1 [67] [68] in oviducts of IL-17-/- mice at day 6 (data not shown) while iNOS and CD86 transcripts were higher in WT mice compared to IL-17-/- mice, findings consistent with an M2 bias early in infection of the IL-17 knockout mice. IL-17 has been shown to synergize with IFNγ to upregulate iNOS and NO production in both epithelial cells and macrophages to inhibit *C. muridarum* growth, which could explain the higher iNOS expression in WT oviducts. It will be important to isolate pure macrophage populations from the reproductive tracts of both strains to confirm this potential bias. Another potential explanation is that the IL-17ra-/- mice were on a C57 background while the IL-17-/- mice in our study were on a BALB/c background. Differences in the course and outcomes of *C. muridarum* genital infections, including the time to clearance, the development of hydrosalpinx and infertility and the Th1/Th2 bias [69] have been observed in different strains of mice [70,71]. Similar strain-dependent differences in weight loss, neutrophil influx and chlamydial tissue burden in lungs are also seen following *C. muridarum* pulmonary infection of mice [72].

IL-17 has indeed been implicated in protection against chlamydial pulmonary infections [49] as well as genital tract infection. Antibody-mediated neutralization of IL-17 during a *C. muridarum* pulmonary infection resulted in greater weight loss, higher organism growth and more severe lung pathology compared to mice that received isotype-control antibody [73], suggesting that IL-17 is important for resolution of pulmonary chlamydial infection. This study also showed that neutralization of IL-17 reduced the capacity of dendritic cells to elicit protective Th1 immunity, and that these DC were more likely to induce Th2 immunity [73]. IL-17 also synergizes with IFNγ to increase iNOS and NO production by macrophages to inhibit *in vitro* growth of *C. muridarum* [55] and *in vivo* neutralization of IL-17 reduced iNOS expression in mouse lung, which resulted in greater *C. muridarum* burden. Together, these studies suggest that IL-17, together with IFNγ, is important for macrophage activation, most likely M1 macrophages, to clear pulmonary chlamydial infection. Collectively, these studies suggest that the mechanisms of clearance of *C. muridarum* differ between the lung, where IL-17 is important, and the genital tract, where both infection and pathology are less severe in the absence of IL-17.

In summary, our data show that IL-17 has contrasting effects on the course of a natural *C. muridarum* infection and the development of vaccine-induced immunity. In the absence of IL-17 the course of a natural infection is significantly attenuated and hydrosalpinx fails to develop. This is likely due to a combination of the greatly reduced influx of inflammatory cells in IL-17-/- mice together with the development of higher levels of *Chlamydia* -neutralizing antibody. However, in the absence of IL-17, immunization protocols that protect WT mice against infection and inflammation are ineffective due to the greatly reduced production of IFNγ and protective Th1 immunity. These results highlight the diverse roles of IL-17 and the plasticity of Th17 cells and suggest that understanding the pro-inflammatory versus protective mechanisms elicited by this cytokine will be key to the development of a successful chlamydial vaccine. We would strongly suggest however, that potential human vaccines must not enhance the recruitment of inflammatory cells into oviducts/fallopian tubes which would likely result in increased inflammatory damage and blockage leading to infertility, the most costly outcome of chlamydial infection. Thus the aim of a vaccine should be to prevent ascending infection, which may not require induction of sterilizing immunity [46].
